# Zhongyong thinking (doctrine of the mean) and internet addiction: The mediation of maladaptive cognition and the moderation of subject

**DOI:** 10.3389/fpubh.2022.1045830

**Published:** 2023-01-26

**Authors:** Hua Wei, Hemuqing Xu, Wu Chen, Lijun Lu

**Affiliations:** ^1^Department of Psychology, Normal College, Qingdao University, Qingdao, China; ^2^School of Educational Science, Xinyang Normal University, Xinyang, China; ^3^School of Marxism, Wuhan University, Wuhan, China

**Keywords:** maladaptive cognition, internet addiction, cognitive behavioral model of pathological internet use, subject, zhongyong thinking

## Abstract

Based on the cognitive-behavioral model of pathological internet use, this study explored the relationship between zhongyong thinking (doctrine of the mean) and internet addiction, and examined the mediation of maladaptive cognition and the moderation of subject. Convenience sampling was used to select 1,518 college students for the questionnaire. The participants were 15–26 years old (*M* = 19.77; *SD* = 1.45), including 776 male and 742 female students. The results showed that zhongyong thinking was significantly negatively correlated with maladaptive cognition (*r* = −0.19, *p* < 0.001) and internet addiction (*r* = −0.14, *p* < 0.001). Maladaptive cognition was significantly positively correlated with internet addiction (*r* = 0.46, *p* < 0.001). After controlling for age, gender, zhongyong thinking negatively predicted internet addiction (*B* = −0.06, *p* < 0.05), maladaptive cognition positively predicted Internet addiction (*B* = 0.45, *p* < 0.001). Zhongyong thinking negatively predicted maladaptive cognition (*B* = −0.19, *p* < 0.001). Moreover, the bias-corrected bootstrapping mediation test indicated that the process by which zhongyong thinking predicted Internet addiction through maladaptive cognition was significant, indirect effect = −0.08, *SE* = 0.01, 95% CI = [−0.11, −0.06]. Subject has no moderating effect on the relationship between zhongyong thinking and maladaptive cognition. The interaction between zhongyong thinking and subject was not a significant predictor of maladaptive cognition *(B* = 0.05, *p* > 0. 05). The present results suggest that zhongyong thinking as a traditional Chinese wisdom can still play an important role in regulating young people's behavior in the digital age.

## Introduction

The well-developed information technology has fully integrated into people's everyday lives. Although the internet brings benefits, it has repercussions especially for young people (1–4), for instance, internet addiction. Internet addition among young people has become a serious world wide public health concern with its prevalence estimated to be 0.8% in Iceland, 6.3% in China, and 22.2% in Iran (5, 6). An increasing body of research has revealed that young people who are addicted to the internet are more likely to experience physical and psychological difficulties (7–10). Specifically, young people's higher score of internet addition is positively associated with theirsleep disturbance, anxiety, depression and loneliness (7, 9, 10). Therefore, it is significant to explore and test the factors that influence internet addiction.

Previous studies have found that both environmental factors (e.g., school, family, social support, and peer interactions) and individual factors (e.g., personality, emotion, and cognition) have an impact on internet addiction (6, 11, 12). Although previous studies have been copious, few studies have examined internet addiction in relation to zhongyong thinking (doctrine of the mean), a particular way of thinking within the circle of Confucius culture (13, 14).

Zhongyong thinking indicates the tendency to consider things from different perspectives, take tactics to coordinate with other people and try to evade potential conflicts with others (15, 16). Researchers have created three inter-correlated dimensions of zhongyong thinking: multi-thinking, holism, and harmoniousness (15). Multi-thinking indicates the tendency to take manifold perspectives into consideration before making a decision in a discussion group. Holism indicates the tendency to balance between external information and internal needs and harmoniousness is using indirect interpersonal social skills to express their opinions so as not to cause conflicts. Since zhongyong thinking derives from traditional Confucius culture, it is a cultural commonality in traditional Chinese society. Although the contemporary society pools a mixture of western and Chinese culture, zhongyong thinking still epitomizes Chinese people's group thinking, determining people's attitude, way of doing and decision making. Nevertheless, the degree of holding this thinking style is of individual difference, meaning that some people may be more inclined to take up this thinking style than others.

Zhongyong thinking is specifically important for young people at university, helping them adapt more easily to campus life. Empirical studies have found that individuals high in zhongyong thinking demonstrate better social adaption, including mental health, social skill and academic achievement (16–20). Young people will encounter more complicated social situations and more diverse peer types in university compared with their lives in basic educational system. University students come from different places across the country and thus their lifestyle, thinking style and value can remain divergent from one another. These differences may increase interpersonal conflicts and disrupt interpersonal relationships (21, 22). However, individuals high in zhongyong thinking may tolerant or accept these differences by reconsideration, find a balance between self needs and other needs and thus harmoniously manage the potential interpersonal tensions. Additionally, Chinese university students may have academic and career stress while zhongyong thinking can arm students with better coping skills and emotional regulation ability (20), which protect them from developing psychological issues when facing multiple stress sources.

Previous studies have shown that young people who have adaptive issues may treat the internet as a shelter, which increases the risk of they developing internet addiction (6, 11). As zhongyong thinking can help young people adapt better to campus life, individuals who score high on zhongyong thinking may have lower degree of internet addiction. Empirical evidence has also found a significantly negative correlation between zhongyong thinking and internet addiction among college students, which suggests that zhongyong thinking can be a protective factor for internet addiction (13, 14). Previous research has also found that zhongyong thinking can impact on internet addiction through the mediations of social interaction and negative emotions (13, 14). Although existing studies have partly explained why zhongyong thinking is associated with internet addiction, very few studies examine the topic in terms of cognitive characteristics.

According to the cognitive-behavioral model of pathological internet use, individual characteristics influence internet addiction through the mediating role of maladaptive cognition (23). Maladaptive cognition indicates the distorted perception that online world is better and happier than offline world and the online self is more excellent than the real self. However, individuals high in zhongyong thinking tend to adapt better to real-life situations than others (13, 14, 19, 20). Those well-adapted individuals have less maladaptive cognition, leading to a lower level of internet addiction (24). Therefore, maladaptive cognition may mediate the relationship between zhongyong thinking and internet addiction.

Although zhongyong thinking is beneficial for individual adaption, the effect may vary across different individual groups. We speculate that zhongyong thinking may play a more important role for individuals who have more negative emotions, compared to those who have less negative emotions. For example, prior evidence showed that the effect of zhongyong thinking improving task performance behavior presents larger for individuals who suffer more negative emotions (25). In addition, previous research has revealed that arts students experience more negative emotions than science students (26). Therefore, zhongyong thinking may be more helpful for arts students than for science students, as it restrains arts students' maladaptive cognition more strongly.

The present study, therefore, aim to construct a moderated mediation model to explore the relationships between zhongyong thinking and internet addiction. The first step is to examine whether maladaptive cognition mediates the indirect path between zhongyong thinking and internet addiction. The second step is to investigate whether subject moderates the indirect path between maladaptive cognition and internet addiction.

## The relationship between zhongyong thinking and internet addiction

People high in zhongyong thinking usually exhibit competence in regulating their behavior, cognition and emotions. In behavioral terms, zhongyong thinking can contribute to developing innovative behavior, voice behavior, and employee performance (27). In cognitive terms, zhongyong thinking can increase creative thinking (28). In emotional terms, zhongyong thinking can increase emotional regulation and reduce negative emotions (13, 18, 20).

Young people who suffer from internet addiction cannot resists the temptation from the internet and spend excessive time on it, which result in damage to their study and life. Although the internet offers university students tremendous benefits including acquiring knowledge, expanding social circle and amending negative emotions, it brings about drawbacks including damaging physical health, consuming time and damaging real-life social interaction. Zhongyong thinking, nonetheless, emphasizes multiple perspective and holistic view, which may help young people realize both the advantages and disadvantages of the internet. In that case, they would plan their use of the internet more rational, leading to less probability of internet addiction.

Specifically, after considering both the advantages and disadvantages of the internet, young people high in zhongyong thinking may increase their time spent on instrumental matters (e.g., information collecting or online learning) and decrease time on hedonic matters (e.g., playing games or watch online videos). Compared to instrumental use, hedonic use can bring strong pleasure and flow experience, forming a negative reinforcement process on internet addiction. Numerous empirical studies have demonstrated that hedonic internet use can positively predict internet addiction, whereas instrumental use negatively predict it (29–31). Therefore, zhongyong thinking may differently affect internet addiction regarding different types of internet use.

In addition, zhongyong thinking may prompt young people to control the total time they spent on the internet. Excessive internet use may trigger users' background craving of internet, which may increase the risk of internet addiction. Empirical studies have also shown that the time of internet use is positively associated with internet addiction (30, 31). Therefore, zhongyong thinking can influence internet addiction through time spent on the internet. This argument is empirically supported by previous studies (13, 14). Thereby, we proposed.

Hypothesis 1: Zhongyong thinking was negatively associated with internet addiction.

## The mediation of maladaptive cognition

Although a few studies have examined the relationship between zhongyong thinking and internet addiction (13, 14), the mediating mechanisms have not been fully explored. According to the cognitive-behavioral model of pathological internet use, individual characteristics influence internet addiction through maladaptive cognition (23). Therefore, the study will examine the mediation of maladaptive cognition, a concept defined as the perception to consider the online world to be better than the real world (23).

Firstly, zhongyong thinking may decrease maladaptive cognition. On the one hand, young people high in zhongyong thinking are usually well-adaptive in offline world, which makes online world not as attractive as to those who are not so accomplishing in real life. Given their tendency to think from multiple and holistic perspectives and pay much attention to interpersonal harmony, they tend to maintain amiable social atmosphere around acquaintances and often have better interpersonal skills. As a situational factor, social interaction greatly impacts on cognition (32). In a similar vein, how successful individuals' social life in reality and on the internet determine the emergence and persistence of maladaption cognition (33). University life is usually more complicated than individual earlier life regarding the difference in young people' s values, lifestyles, ways of thinking and habits (21, 22). Such heterogeneous situations require young people to have more interpersonal skills and wisdom in dealing with potential interpersonal conflicts. Zhongyong thinking, indeed, is practical in these situations.

On the other hand, young people low in zhongyong thinking, whose social performance in real life is unsatisfactory, may have a chance to achieve social prestige in the online world. Unlike the real world, the online world has the ability to filter out people who are different from the self, rendering possible interactions within people who are highly homogeneous (4). Such similarity in the social environment increases interpersonal attraction and satisfaction (34), drawing these young people into the shelter of the internet. With regard to empirical evidence, researchers have found a negative association between zhongyong thinking and psychopathology, such as anxiety and depression (16, 18), which increase maladaptive cognition (23).

In addition, maladaptive cognition has a positive effect on internet addiction. The core features of maladaptive cognition are the overestimation of the virtual world and the devaluation of the real world (23). Individuals with a high level of maladaptive cognition think the virtual world good and comfortable, while the real world is bad and unbearable. Therefore, they will form positive outcome expectations about their online experience and then invest a lot of time and energy into the internet (23). Furthermore, the internet can provide individuals with a lot of content stimulation and various forms of activities which reinforces the use of the internet (23). However, the over-satisfaction in the virtual world may erode the agency and desire for living in the real world. Investing too much time and energy on the internet may leave individuals insufficient resources to fulfill their real-life roles, thereby harming their social, academic and living functions (23). Repeated satisfaction in the virtual world and frustration in the real world may further reinforce maladaptive cognition. Gradually, individuals may lose control of themselves online and their real life will be worsened, which will eventually develop into internet addiction. Masses of empirical studies across culture and age have found a positive association between maladaptive cognition and internet addiction (35–37). Accordingly, we proposed.

Hypothesis 2: Maladaptive cognition plays a mediating role in the relationship between zhongyong thinking and internet addiction. Zhongyong thinking is positively correlated with maladaptive cognition while maladaptive cognition is negatively correlated with internet addiction.

## Moderation of subject

Considering that subject is a prominent identity for university students, we assume that subject may be a moderator on the relationship between zhongyong thinking and maladaptive cognition. Some evidence in previous literature indicates that science students score higher than arts students on the openness dimension in the personality scale (38), which supports our assumption. Openness motivates young people to broaden their horizons by seeking out novel knowledge and experience which often challenge their current opinions. As a result, they are inclined to accept difference and have less prejudice and discrimination and more likely to form positive belief about people from outgroups (39–41). Therefore, university students high in openness are more likely to accept different values and lifestyles, resulting in less interpersonal conflicts with people who hold different opinions. To sum up, openness plays a similar role as zhongyong thinking in coping potential interpersonal conflicts. Although zhongyong thinking reduces maladaptive cognition through decreasing interpersonal conflicts, the effect is weaker among people high in openness.

As openness has been adequate in helping science students to manage their social life, the effect of zhongyong thinking on science students' maladaptive cognition may not be as stronger. In contrast, arts student whose openness trait is less than science students, are more likely to be involved in interpersonal troubles. Therefore, zhongyong thinking can be more effective for arts students in terms of restraining maladaptive cognition.

Based on the above argument, we propose hypothesis H3.

Subject moderates the relationship between zhongyong thinking and maladaptive cognition. Zhongyong thinking style has a greater impact on maladaptive cognition for arts students than for science students.

## The present study

The purpose of this study was threefold. First, we examined whether zhongyong thinking predicts adolescent internet addiction by proposing Hypothesis 1.

Hypothesis 1: Zhongyong thinking was negatively associated with internet addiction.

Subsequently, we tested a mediator model to examine how zhongyong thinking is correlated to internet addiction by proposing Hypothesis 2.

Hypothesis 2: Maladaptive cognition plays a mediating role in the relationship between zhongyong thinking and internet addiction. Zhongyong thinking is positively correlated with maladaptive cognition while maladaptive cognition is negatively correlated with internet addiction.

Finally, we examined whether subject moderated the relationship between zhongyong thinking and maladaptive cognition, by advancing Hypotheses 3.

Hypothesis 3: Subject moderates the relationship between zhongyong thinking and maladaptive cognition. Zhongyong thinking style has a greater impact on maladaptive cognition for arts students than for science students.

**Figure 1:**
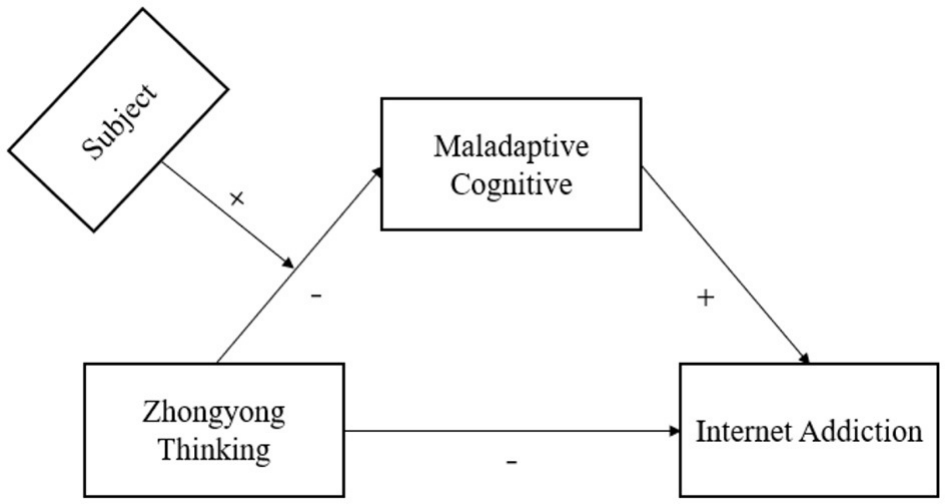
The hypothetical model.

Please see the hypothetical model in Figure 1.

## Materials and methods

### Participants

A convenience sample was taken and 1,575 college subject were selected from five universities in Henan province, one university in Jiangsu, one university in Shandong and one university in Anhui. Five participants refused to submit their answers. A total of 1,518 valid questionnaires was obtained after deleting the 5 withdrawers' answers along with 52 regular and incomplete answers. The invalid answers were deleted according to the order as follows: 34 regular answers and 23 incomplete answers, including 1 from gender, 1 from age, 1 from subject, 11 from zhongyong thinking, 6 from maladaptive cognition and 3 from internet addiction. Subject were aged 15–26 years (*M* = 19.77; *SD* = 1.45), of whom 776 were male and 742 were female; 403 were arts students and 1,115 were science students; 710 were freshmen, 413 were sophomores, 292 were juniors, and 103 were seniors.

## Measures

### Zhongyong thinking

Zhongyong thinking was measured by the Chinese version of the Zhongyong thinking Style Scale (15). This scale consists of 13-items rated on a five-point scale (1 = “strongly disagree,” 7 = “strongly agree”). Examples of items were as follows “I am used to think about things in multiperspectives,” “I seek a compromise between my opinions and others,” “When making decisions, I tend to consider the overall atmosphere and try to maintain harmony.” The scale has shown acceptable reliability and validity (16, 18, 20, 25). In this study, the Cronbach's alpha of the total scale was α = 0.94.

### Maladaptive cognition

Maladaptive cognition was measured by a maladaptive cognition scale in Chinese (23, 42). This scale consists of 4 items, for example, “I am worthless offline, but online I am someone.” Participants evaluated the items on a 5-point Likert scale ranging from 1 = *Never* to 5 = *Always*. The scale has been commonly used among Chinese participants and has good reliability and validity (33, 42, 43). In this study, the Cronbach's alpha of the total scale was α = 0.92.

### Internet addiction

Internet addiction was measured by the Chinese version of the internet addiction scale (44), which includes 8 items. A 5-point Likert scale was adopted (1 = *not at all true* to 6 = *completely true*). The scale has been demonstrated to has good reliability and validity on Chinese samples (13, 14). In this study, the Cronbach's alpha of the total scale was α = 0.89.

### Statistical analyses

First, before the test, we employed several approaches toreduce common method bias, including participant anonymity, rearrangement, and reverse expression of items. In addition, we employed Harman's single factor test to determine whether common method bias exists in this study. The results showed a multiple-factor structure, and that the largest loading factor only accounted for 33.23% of the total variance, far less than the 40.00% threshold, suggesting no significant common method bias in this study (45).

Second, descriptive analysis was performed to examine the participants' characteristics regarding the studied variables, and Pearson correlation analysis was performed to examine the correlations between variables.

Third, the present study used PROCESS version 3 to test the mediating and moderated model (46). Given that PROCESS Marco does not provide standardized regression coefficients, we calculated z-scores before data analyses. We generated 5,000 bootstrapped samples to approximate the confidence interval (CI) of the indirect effect based on the original sample (*n* = 1,518). A 95% bias-corrected accelerated CI without zero indicates statistical significance. In addition, the age and gender of the participants were controlled in the analyses.

## Results

### Examination of the measurement model

We assessed the discriminant and convergent validity of the measurement model following previous research (47). We evaluated the convergent validity for all latent measures using satisfactory standardized factor loadings. All factor loadings ranged from 0.56 to 0.90, reaching the criterion of 0.40 or above (48). We included three criteria for the evaluation of convergent validity: Cronbach's alpha value, composite reliability (CR), and the average variance extracted (AVE). As demonstrated in Appendix, the alpha values for all variables ranged from 0.89 to 0.94, reaching the criterion of 0.70 or above (49). The CR values ranged from 0.89 to 0.94, agreeing with Hair et al. (47) criterion of 0.70 or above (47). Finally, the AVE values of all variables ranged from 0.51 to 0.74, reaching the criterion of 0.36 or above (50). Therefore, all of the results satisfied the criteria for convergent validity. Additionally, we evaluated divergent validity using the AVE–SV comparison (49). As demonstrated in Appendix, all of the square roots of the AVE were higher than the correlation among the constructs, satisfying the criteria for divergent validity (49).

### Preliminary analyses

Table 1 presents the Pearson correlations, means, and standard deviations of all variables. As Table 1 indicates, zhongyong thinking was negatively correlated with maladaptive cognition and internet addiction. Maladaptive cognition was positively correlated with internet addiction.

**Table 1:** Correlation coefficients, means, and standard deviations of variables.

	***M ±SD***	**1**	**2**	**3**
1. Zhongyong thinking	5.08 ± 0.96	—		
2. Maladaptive cognition	1.31 ± 0.58	−0.19^***^	—	
3. Internet addiction	2.37 ± 1.01	−0.14^***^	0.46^***^	—

*N* = 1,518, ^***^p < 0.001.

### Testing a moderated mediation model

The analysis of mediated effects with moderation is performed in two steps, first testing the simple mediated model and then the mediated model with moderation.

**Figure 2:**
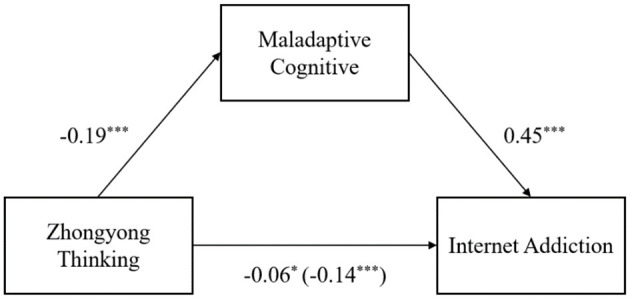
The Integrated Model. The values in parentheses are the total effect of Zhongyong thinking on Internet addiction. Path values are the path coefficients, *p < 0.05, ***p < 0.001.

As a first step, the simple mediation model is tested first. Among the mediated models with adjustment, the simple mediated model is the benchmark model, so it is tested first (46). We used Model 4 of PROCESS (46) to examine the possible association between zhongyong thinking and internet addiction as well as the possible mediating effect of maladaptive cognition. The results of the mediation analysis are presented in Table 2 and Figure 2. After controlling for age, gender, we first found that Zhongyong thinking negatively predicted internet addiction *B* = −0.06, *p* < 0.05, maladaptive cognition positively predicted internet addiction, *B* = 0.45, *p* < 0.001 (Equation 1). Second, zhongyong thinking negatively predicted maladaptive cognition, *B* = −0.19, *p* < 0.001 (Equation 2). Finally, the bias-corrected bootstrapping mediation test indicated that the process by which zhongyong thinking predicted internet addiction through smaladaptive cognition was significant, indirect effect = −0.08, *SE* = 0.01, 95% CI = [−0.11, −0.06]. The results of the mediation analysis support H1 and H2.

**Table 2:** Mediation of maladaptive cognition.

	**Equation 1**		**Equation 2**	
	**(Criterion** = **Internet addiction)**		**(Criterion** = **Maladaptive cognition)**	

**Predictors**	***B***	***t***	***B***	***t***
Gender	0.08	1.77	−0.23	−4.68^***^
Age	−0.02	−1.46	−0.003	−0.17
Zhongyong thinking	−0.06	−2.53^*^	−0.19	−6.78^***^
Maladaptive cognition	0.45	18.26^***^		
*R* ^2^	0.22		0.05	
*F*	99.44^***^		21.01^***^	

^*^p < 0.05, ^***^p < 0.001.

In the second step, the test has a moderating mediating role. Since the moderating variable regulates the first half of the mediating effect, mode 7 is chosen for testing. The results of the moderation analysis can be found in Table 3. Regression analyses indicated that zhongyong thinking negatively predicted maladaptive cognition (*B* = −0.19, *p* < 0.001) and subject was not a significant predictor of maladaptive cognition (*B* = −0.04, *p* > 0. 05), while the interaction between zhongyong thinking and subject was not a significant predictor of maladaptive cognition *(B* = 0.05, *p* > 0. 05), with a 95% confidence interval of [−0.08, 0.19] and contain 0. The results indicate that subject has no moderating effect on the relationship between maladaptive cognition and zhongyong thinking, and hypothesis H3 was not tested.

**Table 3:** Tests for mediating effects with moderation.

	**Equation**	
	**(Criterion** = **Maladaptive cognition)**	
**Predictors**	***B***	***t***
Gender	−0.24	−4.42^***^
Age	−0.003	−0.17
Zhongyong thinking	−0.19	−6.78^***^
Subject	−0.04	−0.66
Zhongyong thinking × subject	0.05	0.76
*R* ^2^	0.05	
*F*	13.25^***^	

^***^p < 0.001.

## Discussion

### The relationship between zhongyong thinking and internet addiction

This study found a negative relationship between zhongyong thinking and internet addiction, which is consistent with the results in previous studies (13, 14). The present results suggest that zhongyong thinking as a traditional Chinese wisdom can still play an important role in regulating young people's behavior in the digital age.

Although this study sampled from Chinese population, the results can communicate to a larger audience as internet addiction has long been a social and educational problem in other Asian countries deeply influenced by Confucius culture (17, 27, 51), such as Japan, Korea and Malaysia (6, 8). Empirical studies have demonstrated that zhongyong thinking has a significantly positive impact on mental health of young people from East Asia (17), suggesting that the present findings may adapt to these countries.

Moreover, this study may provide a better knowledge of intervening internet addiction in western society. Although zhongyong thinking as a concept does not exist in western culture, *social mindfulness* share many similarities with zhongyong thinking regarding theoretical overtones and functioning mechanism (52). For example, the two concepts both focus on social value, underlining the attention on other people's need. Zhongyong thinking can increase social support, decrease interpersonal conflicts and increase prosocial behavior within a group (13, 14, 53). Similarly, social mindfulness also can help individuals achieve social applaud and trust, which subsequently increases individual prosocial behavior (52, 54). Given these similarities, we speculate that social mindfulness may influence internet addiction.

### The mediation of maladaptive cognition

This present study found that maladaptive cognitive mediated the relationship between zhongyong thinking and internet addiction. Young people high in zhongyong thinking may be more easily to integrate into the social environment of a complex campus and have better social experience in everyday life. In comparison, individuals low in zhongyong thinking are likely to experience frustration in a diverse campus and become overly dependent on the internet to provide social need satisfaction, ultimately increasing the risk of maladaptive cognition and internet addiction.

We introduced a theoretical perspective to explain why zhongyong thinking decreases the risk of internet addiction and illustrate its mediating mechanism. From the perspective of cognitive-behavioral model of pathological internet use, distal individual characteristics can influence internet addiction through proximal factors, for example, maladaptive cognition (23). This model provides us a way of understanding the functioning mechanism underlying the relationship between zhongyong thinking and internet addiction, which has barely been investigated in previous research. Past research examining the mediation of the relationship between zhongyong thinking and addiction has mostly focused on distal factors such as social support, peer conflict and loneliness (13, 14) while proximal factors were hardly discussed. As a proximal factor, maladaptive cognition is more closely linked to internet addiction in terms of etiology compared with previously studied variables. Examining proximal factors can deepen our understanding regarding how zhongyong thinking affects internet addiction and offer suggestions for relevant interventions.

Intervention practitioners often choose to consider a mediating factor instead of directly intervening the dependent variable. With respect to internet addiction, interventions for maladaptive cognition has advantages over social support, peer conflict, and loneliness. Additionally, maladaptive cognition has been demonstrated to be effective in intervening internet addiction and existing maladaptive cognitive intervention programs for internet addiction are systematic and mature and conducive to widespread application (55). Moreover, the effect of interventions for maladaptive cognition was more individually based, whereas the effect of interventions for social interaction and loneliness also counts on the social environment.

The cognitive-behavioral model of pathological internet use emphasizes the importance of maladaptive cognition and the mediating role in the relationship between individual characteristics and internet addiction. Although copious previous studies have demonstrated the model, most of them focus on individual characteristics of cultural universality, for example, personality and social anxiety (37, 56, 57), and only one study have noticed individual characteristics of cultural particularity (58). The present study expanded the cultural scope of the model by investigating zhongyong thinking, a characteristic of traditional Chinese culture, influencing Internet addiction through maladaptive cognition. Future research could further examine whether there are other culturally distinctive factors which influences internet addiction and maladaptive cognition, further enhancing the cultural depth of the cognitive-behavioral model of pathological internet use.

### Moderation of subject

The results of moderation analysis indicated that subject can not significantly moderate the relationship between zhongyong thinking and maladaptive cognition, which failed to demonstrate our Hypothesis 3. For both arts students and science students, zhongyong thinking can reduce their maladaptive cognition. Our previous assumption was that the effect might be significantly weaker for science students than for arts students because empirical research found that science students scored higher on openness scale and had less interpersonal conflicts, which protected them from developing maladaptive cognition.

However, science students' lower scores on extroversion and received social support (38, 59) may play a confounding role in our hypothetical moderation model, leading zhongyong thinking an important factor to reduce maladaptive cognition both for science students and arts students. Individuals low on extroversion rarely start a social conversation and they are less likely to reveal themselves during social interaction (60, 61). These traits reduce their odds of obtaining social support (59). Therefore, in light of extroversion, science students may be more difficult than arts students to receive social support. Zhongyong thinking, however, bears the features of taking others' needs into consideration and emphasizing the harmony of relationship. With these traits, science students will be more likely to receive social support (13), which subsequenty reduces their maladaptive cognition (62).

### Limitations and implications

The first limitation lies on the fact that we merely studied young people from mainland China, rendering it impossible to generalize the results to a more general population. As zhongyong thinking also exists or even prevails in other east Asian countries (17, 27, 51), future studies can test the results among young people from these places. The second limitation is the homologous data, which may cause shared variance among the researched variables and exaggerated the effects between variables. Therefore, researchers can collect data from multiple sources of participants, for example, parents and peers in the future. The third limitation is the cross-sectional design of the current study, which renders it impossible to draw casual conclusions. Longitudinal studies can be conducted to illustrate the directions between variables in this research.

More practical implications could be drawn from this study. First, the result that zhongyong thinking can be effective in reducing internet addiction suggests that practitioners intervene in internet addiction by strengthening zhongyong thinking among college students. Although there are many interventions and programs for internet addiction to date (55), hardly any of them involves Chinese cultural elements. According to an intervention study, mental health and suicide intervention programs based on zhongyong thinking have shown to be effective. Compared to general group counseling, group counseling based on zhongyong thinking can be more effective in reducing depression among college students (16). Dialectical behavior therapy in relation to zhongyong thinking has also been demonstrated to be effective in alleviating participants' psychological symptoms (63).

## Data availability statement

The raw data supporting the conclusions of this article will be made available by the authors, without undue reservation.

## Ethics statement

The studies involving human participants were reviewed and approved by the Research Ethics Committee of School of Educational Science, Xinyang Normal University. Written informed consent to participate in this study was provided by the participants' legal guardian/next of kin.

## Author contributions

HW, WC, and HX designed the work. HW, HX, and LL provided the method. HW, WC, and HX collected the data and analyzed the data results and drafted the manuscript. WC and LL revised the manuscript. All authors contributed to the article and approved the submitted version.

## Appendix

**Table A1:** Validity and reliability of latent variable constructs.

**Construct**	**Standardized factor loading**	**Cronbach's alpha**	**Construct reliability (CR)**	**AVE**
Zhongyong thinking		0.94	0.94	0.55
Item 1	0.56			
Item 2	0.70			
Item 3	0.63			
Item 4	0.80			
Item 5	0.84			
Item 6	0.83			
Item 7	0.78			
Item 8	0.79			
Item 9	0.71			
Item 10	0.72			
Item 11	0.73			
Item 12	0.75			
Item 13	0.74			
Maladaptive cognition		0.92	0.92	0.74
Item 1	0.80			
Item 2	0.89			
Item 3	0.90			
Item 4	0.85			
Internet addiction		0.89	0.89	0.51
Item 1	0.76			
Item 2	0.80			
Item 3	0.78			
Item 4	0.76			
Item 5	0.64			
Item 6	0.62			
Item 7	0.64			
Item 8	0.68			

Note. N, 1518. AVE,average variance extracted.

**Table A2:** The item of each variable.

**Variable**	**Item**
Zhongyong thinking	1. I give consideration to opinions which are different from mine during discussing.
	2. I am used to think about things in multiperspectives.
	3. Before making decisions, I give ear to all opinions.
	4. I give a comprehension consideration while making decisions.
	5. I will find a conclusion which can reach a consensus when different opinions exist.
	6. I seek a compromise between my opinions and others'.
	7. I make adjustment to my previous thought after taking other's opinions into consideration.
	8. I expect to reach a consensus after discussing.
	9. I integrate my opinions into others'.
	10. I tend to express conflicting ideas in a tactful way.
	11. When making decisions, I try to make the minority to obey the majority in a harmony way.
	12. When making decisions, I tend to consider the overall atmosphere and try to maintain harmony.
	13. When making decisions, I tend to adjust my expression in order to maintain harmony.
Maladaptive cognition	1. The Internet is the only place I am respected.
	2. I am worthless online, but online I am someone.
	3. Nobody loves me offline.
	4. The Internet is my only friend.
Internet addiction	1. Feel preoccupied with the Internet (think about previous on-line activity or anticipate next on-line session).
	2. Feel the need to use the Internet with increasing amounts of time in order to achieve satisfaction.
	3. Repeatedly tried to control, reduce, or stop accessing the Internet, but without success.
	4. Feel restless, moody, depressed, or irritable when attempting to cut down or stop Internet use.
	5. Stay on-line longer than originally intended.
	6. Jeopardized or risked the loss of significant relationship, job, educational or career opportunity because of the Internet.
	7. Lied to family members, therapist, or others to conceal the extent of involvement in the Internet.
	8. Use the Internet as a way of escaping from problems or of relieving a dysphoric mood (e.g., feelings of helplessness, guilt, anxiety, depression).
